# Communication

**Published:** 1900-02

**Authors:** Wm. Jopling


### COMMUNICATION.

---

EDITOR OF THE JOURNAL OF COMPARATIVE MEDICINE, ETC.

DEAR SIR: IN a number of the JOURNAL appeared the following question: "Why does not someone in Michigan stand up and defend the new veterinary law in that State from the open attacks made upon it by members of the profession in that Commonwealth?"

Perchance, as President of the Michigan Veterinary Medical

Association, it properly falls to my lot to make reply to this query, which I will endeavor to do as briefly as possible.

I am not aware of any open attacks having been made on our veterinary act by any recognized members of the profession in Michigan.

This simple statement might suffice, but it may not be out of place to call attention to the probable cause of the above question appearing in the JOURNAL.

In another number appears an editorial on Michigan's veterinary act. The editor criticises the bill somewhat severely. It is not my purpose to defend the bill. It does not require very close inspection to see that it might be improved, particularly from a veterinarian's point of view. But are there not "others?" Let me quote from an article in the JOURNAL: "Those who have fought the legislative battles, national and State, have learned the one bad fact, that the entering wedge of legislation on any new lines is the most difficult to obtain, and only gained after the hardest and most conscientious work. Its after-amendment and correction are yielded readily compared with the difficulties encountered at every step of new legislation. In these modern times the pit-falls of any proposed legislative measures are so numerous that only those who have directed those battles and tented or lived on the legislative field can foresee them."

The veterinarians in Michigan who have sought legislation have found this only too true, but hope that the act they have succeeded in making a law, if only an entering wedge, may pave the way to after-amendment and correction.

One of the several pit-falls that has confronted our committees on legislation is a certain garrulous individual hailing from Grand Rapids, and apparently the writer of a communication which appeared in a back number of the JOURNAL over the signature of the Grand Rapids Veterinary College. A perusal of the article would seem to be all that is necessary to condemn the writer as being what he is, not only a quack, but a chief among quacks, whose quacking might well make the duck family turn green with envy. Like too many people who cannot resist the wiles of a street fakir, but fall victims to their glibness, the average legislator is prone to listen to the vaporings of such characters as the one appearing under the pseudonym of the Grand Rapids Veterinary College.

Not being a member of the Committee on Legislation, I cannot speak from personal knowledge, but I am informed by the committee who had the bill in charge that said bill passed the lower house containing a provision requiring colleges that were to be recognized to come up to the standard of the American Veterinary Medical Association, and when the bill reached the Senate the now dean of the Grand Rapids Veterinary College made such strong objections to that provision that the committee, in order to not sacrifice the measure entirely, agreed to an amendment which makes the bill as it now appears.

The writer of the editorial in the May number of the JOURNAL perhaps, not being aware of these facts, would make the criticism appear more just. I could write much more along this line, but I promised to be brief, and may have trespassed too much now on your valuable space. If any of my points are not well taken I am open to correction, but request that I may be excused from replying to effusions emanating from the Grand Rapids mill.

Respectfully yours,

WM. JOPLING, V.S.

